# A wireless power beaming system using Y shaped reflectarray and rectenna achieving 8.33% power transfer efficiency

**DOI:** 10.1038/s41598-025-00390-y

**Published:** 2025-05-21

**Authors:** Sunanda Roy, Nabanita Saha, Ifana Mahbub

**Affiliations:** The University of Texas at Dallas, 800 W Campbell Rd, Richardson, TX 75080 USA

**Keywords:** Engineering, Electrical and electronic engineering

## Abstract

In this paper, the design and implementation of a wireless power transfer system using an ultrawideband (UWB) circularly polarized multilayer reflectarray antenna (RA) is proposed. This novel system is the first of its kind to be demonstrated in a distributed transmitter (TX) configuration, presenting enhanced efficiency and versatility. The RA is comprised of novel unit cells of “*Y*” shape, where a smaller arm is connected with a vertical arm at a 45° tilted angle, and a rectangular shape is incorporated in the middle layer that contributes to enhancing the gain over the frequency band. The proposed RA operates in the ultra-wideband (UWB) frequency bands (5 to 10 GHz), and it uses a horn antenna as a feed. The *Y*-shaped UWB featured non-uniform element spacing over the active area and a rectangle-shaped middle layer, achieving the realized gain and aperture efficiency enhancement. The antenna achieved a peak realized gain of 21.261 dBi with a 3 dB gain bandwidth (BW) of 18.2%, and a 3 dB axial ratio (AR) BW of 63.11% for transmit antenna (TA) operation. In the proposed distributed WPT system, the spacing between several TXs is optimized in such a way that they are able to generate a collimated beam simultaneously. The achieved Power Transfer Efficiency for different distributed WPT configurations involving one, two, and three transmitters with a single receiver system is 5.03%, 8.33%, and 8.11%, respectively, at a distance of 1.7 m with an antenna spacing of 660 mm. The proposed well-performed WPT system is a promising candidate for long-distance power beaming.

## Introduction

Reflectarray (RA) antennas are extremely flexible and simple to design, as there is no requirement for a feeding network circuit. The BW of the unit cell of an RA can be increased by creating multilayer reflectarrays^1^, single-layer multi-resonant components^2^, and subwavelength cells^3^. The utilization of these particular arrangements, which involve decreasing the time period, facilitated a significant enhancement in the BW, reaching up to $20\%$, for a reflectarray with a diameter of approximately $10\lambda$. For these particular antenna’s diameter, the differential spatial phase delay has no appreciable impact on the BW. The prior works have demonstrated the feasibility of using impedance-matched elements based on dielectric metamaterials^4^, the Bessel filter method^5^, and a transformation optics (TO) strategy^6^ to increase the BW of RAs. However, there are still some disadvantages to these designs, such as the difficulty in element shape or prototype fabrication, as well as the insufficient consideration of the mutual coupling effect in the unit cell simulation and interlaced element grid, which degrades the RA’s gain performance and grating lobe in the upper-frequency range. To achieve phase compensation, several techniques have been proposed in prior works, such as variable element size^7^, and size-varying dielectric material perforation^8^, respectively. For dual-band circularly polarized RA, ring, patch element, and delay lines and circular rings are used^9,10^. There are mainly two shortcomings of prior proposed techniques, and they are- (1) the requirement to limit the size of the lower band RA elements in order to avoid obstructing the upper band RA and (2) the emergence of grating lobes at the upper band due to the large cell size of lower band components.

Several formulations have been proposed in prior works to forecast the directivity of an RA accurately and, subsequently, its aperture efficiency based on the feed radiation pattern^11^. The aperture efficiency of an RA is typically controlled by it’s feed antenna, and hence high gain, circularly polarized, and miniature size feed antenna design is a primary concern during the reflector array’s design process^12,13^. Since spillover and field taper efficiency values determine the aperture efficiency limit, optimization of these values in the feed is crucial to increase the limiting aperture efficiency. CP antennas are often used in satellite communications because of their ability to mitigate environmental interference. CP antennas offer numerous advantages over antennas utilizing linear polarizations, making them a crucial technology for a wide range of wireless systems such as satellite communications, mobile communications, global navigation satellite systems (GNSS), wireless sensors, radio frequency identification (RFID), Direct Broadcasting Service (DBS) television reception systems and so on.

In this paper, a novel ultrawideband unit element is proposed, which is used to design multilayer ultrawideband RA. The contributions of this work are: (1) the design of a simple, compact, and straightforward *Y* shaped ultrawideband unit element which achieves an ultrawideband performance (5 to 10 GHz, FBW of $63.11\%$) and $54.75\%$ radiation efficiency which is a $20\%$ improvement than some of the state-of-the artworks^14^, (2) the design of a rectangular slotted middle layer which contributes to the overall BW performances, (3) the non-uniform element spacing between each element contributes to an increase in side lobe levels (SLL) and a decrease in the coupling effect between array elements, and (4) the array element achieved a circular polarization performance for 6 and 7.78 GHz frequency bands, and (5) active feeding strategy that is applied through the displacement of the proposed feeding position in front of the RA and achieved wide beam steering coverage (± $60^\circ$).For experimental validation of the *Y*-shaped design concept and operating frequency between 5 and 10 GHz for a complete RA that has been designed, fabricated, and tested.

Distributed Wireless Power Transfer (WPT) systems use multiple transmitters to wirelessly deliver power to a receiver, enhancing power transfer efficiency and coverage. In^15^, the antenna location for downlink distributed antenna systems (DASs) is optimized, but there are some limitations of these prior works. The limitations include lack of scalability, real-time adaptability, and mitigating interference. These limitations hinder the practical implementation of distributed WPT systems in complex and dynamic environments, where precise coordination and energy management are crucial. To address these problems, a distributed reflectarray antenna system is proposed for long-range power beaming to charge unmanned vehicles or drones. It shows better interference suppression ability as the alignment issue is resolved in this work.

**Fig. 1:**
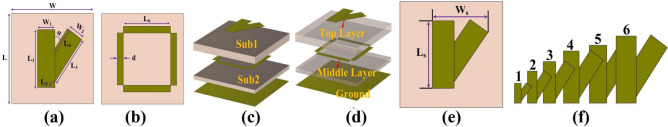
Proposed RA (**a**) single unit element, (**b**) middle layer rectangular slot, (**c**) stack-wise substrates, (**d**) stack-wise layers, (**e**) defined scaling notation of the unit element, and (**f**) six different scaling factors of the unit element.

In summary, the major contributions of this work are as follows:

1. Exhibiting the full wave simulated results for newly designed RA, including radiation patterns, aperture efficiency, frequency responses, and main beam squint.
2. A multilayer RA design concept is introduced to solve the phase range or spatial delay problem of single-layer RA, where phase variation is provided by varying the dimensions $L_s$ and $W_s$ of the unit element.
3. Achieving a higher gain and circular polarization performance through a circular ring-shaped array of elements with non-uniform spacing and a wide steering range by applying active-feeding scenarios.
4. For the very first time, distributed reflectarray antennas are used as TX for long-range power beaming to charge unmanned vehicles or drones.
5. The separation between Txs and their feeding is optimized to prevent any blockage effects, allowing the three Txs to simultaneously function as a collimated beam towards the receiver end.

Section “Design and analysis of reflect array single element” describes the full-wave simulation of the proposed designed *Y*-shaped circular structure RA. The detailed performance analysis of the complete RA is explained, and the effect of the additional layer on the overall gain performance has also been analyzed. The complete performance analysis is demonstrated in terms of aperture efficiency, gain, and radiation pattern in Section “Elevation plane normalized radiation pattern analysis inside the anechoic chamber”, which clarifies the overall achievement by comparing it with several state-of-the-art works. Section “Active feeding strategy with corresponding beamsteering performance” describes the active feeding strategy with corresponding beam steering performance, and Section “The demonstration of the wireless power transfer (WPT) system for different casescenarios” describes the WPT system for various scenarios, including different distances, numbers of Txs, and real receiver (Rx) systems. Finally, Section “Conclusion” concludes this work and provides directions for future research.

## Design and analysis of reflectarray single element

### Geometry of the proposed “*Y*” shaped radiating element

The design of the proposed reflectarrays is based on satisfying certain specifications, such as frequency, bandwidth, polarization, gain, and efficiency. The first stage in the design process of a reflectarray involves the selection of a unit cell capable of linearly spanning the $0^\circ$ to $360^\circ$ range, with modifications in the unit cell optimizing parameter. To fulfil this criterion, it is necessary for the phase of each element to satisfy the following equation^16^.

1 $$\begin{aligned} K_0(R_i-\vec {r_i}.r_b)-\gamma _i= 2N\pi \end{aligned}$$

here $K_0$, $R_i$ are the wave numbers in open space and the distance from the phase center of the feed to the center of element number *i* of the reflectarray, $\vec {r_i}$ is the position vector from the center of the reflectarray surface to the center of an element in the reflectarray. The symbol $r_b$ is the unit vector that indicates the direction of the collimated beam. The *N* is an integer, and $\gamma _i$ represents the phase of the *i*th element. The detailed configurations of the proposed “*Y*” shaped radiating element are depicted in Fig. 1a–d. As for the dielectric substrate, this design uses two different thicknesses for FR4 ($\varepsilon _r$ = 4.3, $tan\delta$ = 0.025), 2.8 mm for substrate 1, and 1.6 mm for substrate 2. The aperture size through which the fields radiate to space can be increased by thickening the radiating patch on both substrates. As the thickness of the substrate rises, the fringing effect causes the distance between the radiating edges to increase. This leads to an increase in the capacitance between the patch and the ground plane, resulting in a decrease in frequency. Resonant Moreover, when the substrate thickness increases, the resonance frequency decreases. This makes it easier to match the impedance of the antenna aperture, which increases antenna bandwidth. The proposed design has three cladding copper layers, referred to as the top, middle, and bottom layers, respectively. The layer-by-layer sandwich structure of the proposed antenna element plays a vital role in increasing the bandwidth itself as well as the overall RA. The phase variation is induced by simultaneously changing the dimensions of the unit element. A comparative analysis between wideband single-layer cell elements, some of which have been investigated in previous research, and multi-layer structures demonstrate that the latter permits more pronounced linear phase variations^17^. A sub-wavelength double-layer reflectarray exhibits a substantial enhancement in bandwidth, with a rise of over $80\%$ compared to a standard single-layer half-wavelength design. The primary reason for the increase in antenna gain is the reduced reflection loss associated with double-layer designs. The proposed single element is shown in Fig. 1 and the Table 1 depicts the dimensions of the various design parameters.

**Table 1:** Notation of the different parameters of the Single element and corresponding optimized values.

Optimized parameters	Notations	Value (mm)	Optimized parameters	Notations	Value (mm)
Substrate length	*L*	30	Width of tilted line	$W_2$	6.24
Substrate width	*W*	30	Length from tilted edge	$L_5$	1.76
Length of vertical line	$L_1$	18.78	Tilted angle	$\alpha$	$35^\circ$
Width of Vertical Line	$W_1$	6.25	Middle layer arm length	$L_6$	20
1st length of tilted line	$L_3$	16.77	Middle layer arm width	*d*	10
2nd length of tilted line	$L_4$	7.85	Length of the scaling arm	$L_s$	18.78
Width of the scaling arm	$W_s$	16.20

**Table 2:** Proposed single element dimensions (length $\times$ width) based on the six different Scaling factors.

S.F.	Length, $L_s$ (mm)	Width, $W_s$ (mm)	S.F.	Length, $L_s$ (mm)	Width, $W_s$ (mm)
1	5.33	4.52	4	12.65	10.71
2	7.11	6.02	5	16.86	14.30
3	9.48	8.03	6	22.49	19.05

*S.F.* scaling factors.

**Fig. 2:**
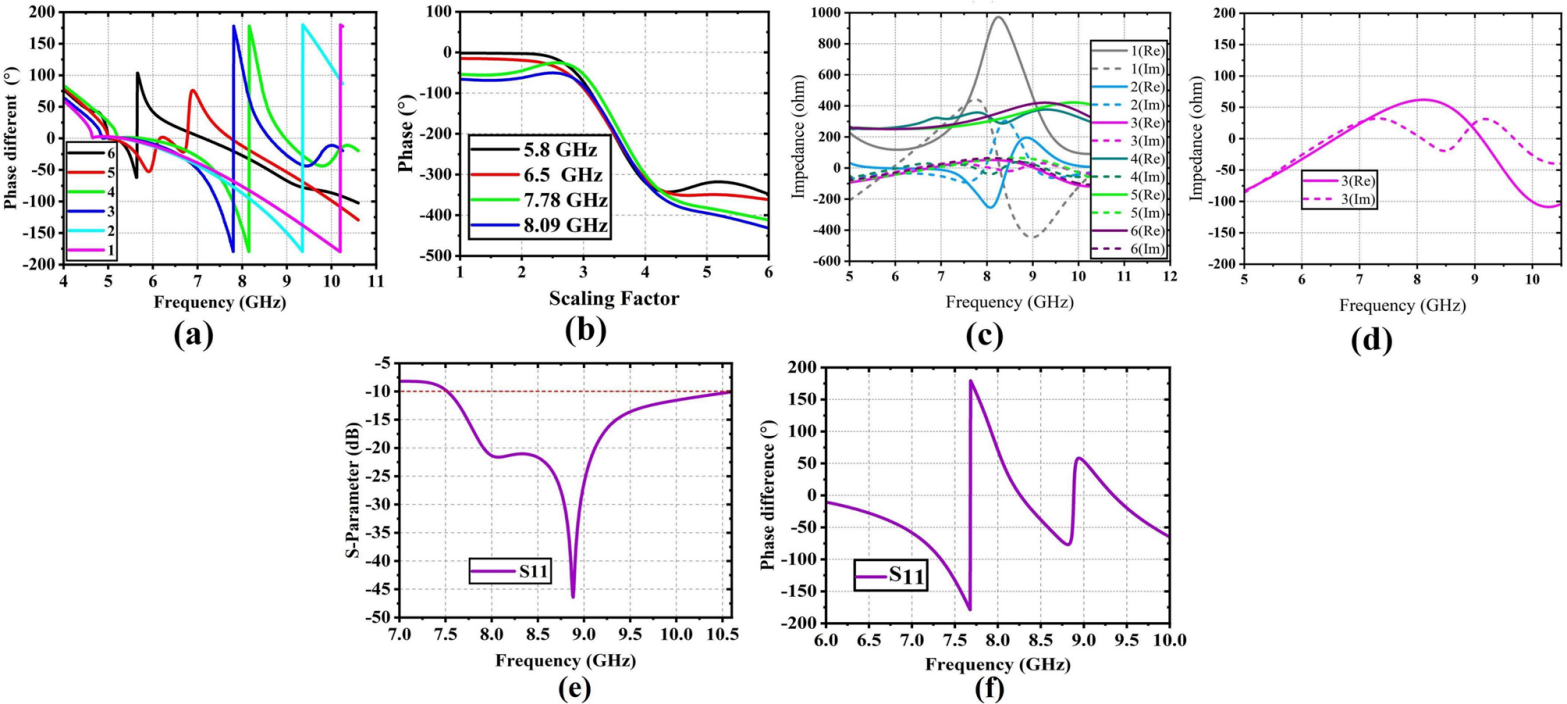
According to the scaling factor (**a**) shifting in resonance frequency by achieving $360^\circ$ phase difference, (**b**) achieved $360^\circ$ phase response, (**c**) characteristic impedance, (**d**) characteristic impedance of the finally selected version for different frequency band, (**e**) S-parameter, and corresponding (**b**) phase response of the single unit cell of the reflectarray.

In order to demonstrate how the element bandwidth influences the RA bandwidth, a relatively small RA that allows for a maximum path loss fluctuation of more than a single $\lambda$ is chosen. The first step is to design a *Y* shaped single element where two lines are connected with a tilted angle ($\alpha$ between them (Fig. 1a). Then, the second step is to design a rectangular shape for the middle layer where each line end edge is connected to each other to pattern a rectangular form (Fig. 1b. The substrate layers, such as sub1 and sub2, are stacked in the third step (Fig. 1c), and the last step (Fig. 1d) explicitly describes the design process for this step, which is layer-by-layer placement with regard to the top or bottom of the substrates. The selection of the single-element dimension is optimized through a scaling factor analysis. The scaling factor analysis is the procedure to determine the final dimension of the proposed single element by analyzing the best performance in terms of the achieved $360^\circ$ phased difference for different frequency bands, the unwrapped values of the achieved phase, and the real and imaginary values of characteristic impedance for different scaling factors. The scaling factors are shown in Fig. 1e,f, and the corresponding optimized values are listed in Table 2. The proposed scaling factor analysis enables a way to determine how the proper dimension of the unit cell covered the frequency range with a constant $360^\circ$ phase difference and achieved a transition phase difference of the ($\le 360^\circ$) for the resonant or target frequency band of the proposed single-unit cell. In Fig. 1f, six scaling factors corresponding to the different dimensions of the proposed single elements are shown. In each case, the tilted angle of the single element remains the same, but the lengths and widths are varied with respect to the scaling factors. The scaling length and width of the proposed single element are denoted as $L_s$ and $W_s$, respectively. Table 2 shows the optimized lengths and widths for the different scaling factors of the single unit element of the RA antenna. The reflection coefficient phase of the unit cell varies as the size of the element varies. Floquet ports and master-slave periodic boundary conditions of the computer simulation technology software are used to analyze the proposed model of the unit element. Figure 2a shows that the maximum phase transition of $360^\circ$ is achieved for 7.78, 8.13, 9.5, and 10.25 GHz, for 3, 4, 2 and 1 scaling factors, respectively. As the dimension of the single element is increased, the phase resonance difference is shifted towards the lower frequency region. It should be noted that, for different frequency bands (i.e., 5.8 GHz, 6.5 GHz, 7.78 GHz, and 8.09 GHz), the achieved maximum phase transition is around $360^\circ$. It can be concluded that the scaling factor 3 is selected due to achieve a $360^\circ$ phase transition, a smaller size for further design of the complete reflectarray system. Figure 2e,f show the S-parameter magnitude and the phase of the $S_{11}$ of the finally optimized unit cell over the frequency range of 7.5 to 10.5 GHz. It can depicted from Fig. 4a that the S-parameter ($\le$ − 10 dB) of the single-unit cell achieved an UWB response from the frequency band of 7.5 GHz to 10.5 GHz. Similarly, Fig. 2f shows the achieved phase resonance over the frequency range of 7.55 to 8.87 GHz. A novel Y-shaped element is used to achieve UWB response of the reflectarray system. The proposed unit cell demonstrates a linear phase response, rendering it a viable contender for applications in the broad X-Ku band. Figure 2a,b represent the achieved phased response for different scaling factors. The quality factor (*Q*), which is calculated from the characteristic impedance of the proposed single unit cell, is one of the determinants of the BW performance of the RA. The characteristic impedance for the different scaling factors is varied, as shown in Fig. 2c, where the difference between the real and imaginary parts over the frequency bands increases as the scaling factors increase. Figure 2d represents the characteristic impedance for the finally selected version (scaling factor 3) of the unit cell for constructing the reflectarray. The equation used for the calculation of the BW is *BW* = ${f_r}$/*Q*: the BW is equal to the resonant frequency ($f_r$) divided by the quality factor (*Q*), where the quality factor (*Q*) is a quantitative assessment of the quality of a resonance circuit. The low *Q* value is caused by a high impedance in series, which leads to a low peak on a wide band response curve. A low resistance connected in series causes a higher value of *Q*, which leads to a narrower BW response. Here, the calculated *Q* values (from Fig. 2d) are 7 and 3 for the frequency bands of 8 and 8.5 GHz, respectively. Based on the impedance response for the scaling factor 3, the value of *Q* factor is less than 7 over the frequency band of operation, which leads to a higher BW response.

**Fig. 3:**
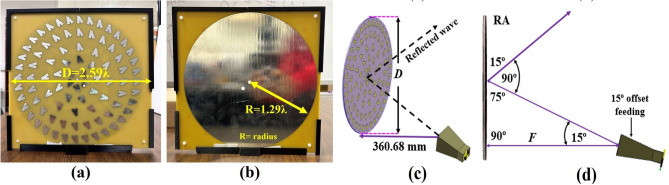
Fabricated RA (**a**) top layer, (**b**) bottom layer, (**c**) CST 3*D* model, and (**d**) angular details of the offset feeding of the RA.

### Prototype implementation and performance analysis of the complete RA

For experimental verification, a prototype of the RA is fabricated as shown in Fig. 3a,b. It shows the proposed RA top and bottom layers and the rectangular slot in between them, which is combined by using plastic screws and 3D printed frame around them. The proposed RA consisting of 86 elements, is designed with a diameter of *D* = 259.56 mm ($2.59\lambda$). An RM-BDHA118-10 horn antenna is used as a feed 360.68 mm (based on optimized *F*/*D* ratio values of 1.4) far away from the surface with a $15^\circ$ offset position, as shown in Fig. 3c,d, respectively. The offset feeding is used to avoid the to reduce the blockage of the beam. The elements distributed across the reflectarray’s surface are not uniform due to both the side lobe level and beamwidth being greatly improved. There are five circular arrays of elements placed across the surface of the antenna. The element spacing varies between the five circular arrays of elements. The element spacing between the innermost arrays of the first circular form is $\lambda /5$ considering the lowest operating frequency, which is 3 GHz. Similarly, for the second, third, fourth, and fifth circular arrays, the element spacing between them is $\lambda /4.3$, $\lambda /3$, $\lambda /3.66$ and $\lambda /5.6$, respectively. This element spacing is optimized based on the reduction in side lobe level and beam width. The non-uniform element spacing between each circular form contributes to an increase in BW and a decrease in the coupling effect between the neighboring array elements.

## Elevation plane normalized radiation pattern analysis inside the anechoic chamber

**Fig. 4:**
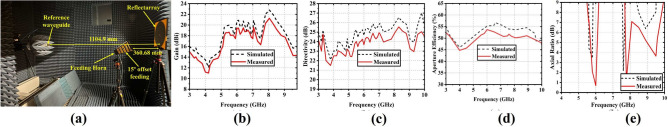
(**a**) Measurement set up inside the anechoic chamber with waveguide and horn antenna, comparison between simulated and measured (**b**) realized gain, (**c**) directivity, (**d**) aperture efficiency, and (**e**) axial ratio of the proposed RA.

Figure 4a depicts the experimental setup which is used to measure radiation patterns of the proposed RA. The *NSI*2000 V4 measurement system is used to measure radiation patterns in the azimuth plane of the RA. The *NSI*–*RF*–*WR*339 (4.07–7 GHz) and *NSI*–*RF*–*WR*340 waveguides (7.05–10.7 GHz) are used as reference antennas that are placed at $14.84\lambda$ (near-field) apart from the RA antenna or AUT (antenna under test). The distance is determined according to the near field region ($\le D^2/\lambda$), where *D* is the diameter of the RA antenna. Figure 4b,c show the comparison between the simulated and measured gains and directivities of the proposed RA. The measured gains at 3.3, 4, 5.5, 6, 6.5, 7.05 8.05, and 8.5 GHz frequency bands are 14.21,11.20,18.685, 19.392, 18.75, 19.752, 21.261, and 19.171 dBi, respectively, whereas the simulated gains are 15.50, 13.87,19.99, 20.81, 19.89, 20, 22.8 and 20.86, dBi, respectively. Within the frequency range of 3 to 9.5 GHz, the difference between the measured and simulated gains ranges from 1 to 3 dB. The measured directivity at X, Y, and Z frequencies are 3.3, 4, 5.5, 6, 6.5, 7.05 8.3, and 8.5 GHz are 24.78, 22.50, 24.02, 23.67, 24.52, 24.95, 26.53 and 25.11 dB, respectively, whereas the corresponding simulated directivities are 25.12, 22.88, 25.26, 24.65, 25.50, 25.81, 26.53 and 26.09 dB, respectively. Blockage and the slight misalignment of the feed could also possibly contribute to introducing an additional loss, causing a discrepancy.

The aperture efficiency of the reflectarray antenna can be presented as follows^18^:

$$\begin{aligned} \eta _{ap}=\eta _{s}\eta _{i} \end{aligned}$$

where $\eta _s$ is spillover efficiency, $\eta _i$ is illumination efficiency, and $\eta _ap$ is aperture efficiency. Aperture efficiency ($\eta _ap$) measures the uniformity of the magnitude distribution of the feed pattern over the surface of the reflectarray as shown in Fig. 4d. The spillover efficiency $\eta _s$ is defined as the percentage of the radiated power from the feed antenna which is intercepted by the reflecting surface. It is notable that the maximum measured aperture efficiency is $54.75\%$ at 6 GHz, whereas the simulated value is $55\%$. Over the frequency range of 3–10 GHz, the aperture efficiency is more than $50\%$ most of the frequency range, which means that the radiation works well. In all other frequency bands, except for 4 to 5.5 GHz and 9 to 10 GHz, the aperture efficiency is less than $50\%$. The simulated and measured axial ratio of the RA is shown in Fig. 4e. It can be observed that the RA can be considered a wave convertor from linear polarization to circular polarization with an axial ratio (AR) that is less than 3 dB in the 6 and 7.78 GHz frequency band. Except for these frequency bands for all other frequency bands, the RA shows linear or elliptical polarization either linear or elliptically polarized. In the proposed single unit cells of the RA, the methods of circular polarized CP radiation mainly use a *Y* shape, where the tilt angle between them is $35^\circ$. Also, the rectangular slot in the middle layer helps to adjust the equal amplitude of the single cell of the RA. The polarization properties of the RA is also controlled by varying the distance of the feeding antenna from the RA surface.

**Fig. 5:**
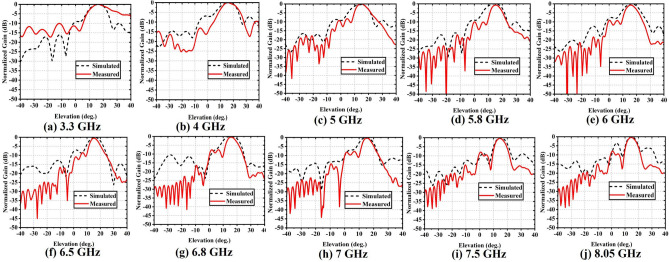
Comparison between simulated and measured normalized gain pattern in the elevation plane at (**a**) 3.3 GHz, (**b**) 4 GHz, (**c**) 5 GHz, (**d**) 5.8 GHz, (**e**) 6 GHz, (**f**) 6.58 GHz, (**g**) 6.8 GHz, (**h**) 7 GHz, (**i**) 7.5 GHz, and (**j**) 8.05 GHz of the proposed RA.

To analyze the radiation properties of the RA antenna in the elevation plane for different frequency bands (i.e., 3.3, 4, 5, 5.8, 6, 6.5, 6.8, 7 7.5, and 8.05 GHz), they are depicted in Fig. 5. The measured and simulated normalized gain patterns in the elevation plane at different frequency bands are reported for $\phi$ = $90^\circ$. It can be seen that the proposed RA produces a collimated beam in the direction of $15^\circ$ due to offset feeding. The SLL of the radiation patterns at different frequencies shows decreasing trends with increasing frequency bands. The simulated SLL of the radiation patterns at 3.3, 4, 5, 5.8, 6, 6.5, 6.8, 7 7.5, and 8.05 GHz are 9.9, 6.25, 8.5, 9.35, 7.35, 6.8, 9.35 7.53, 8.25, and 3.5 dB, while the measured SLL are 11.10, 14.12, 8.21, 10, 8.52, 8.01, 7.5 7.02, 8.21 and 9.25 dB, respectively. Similarly, measured half-power beamwidth (HPBW) at 3.3, 4, 5, 5.8, 6, 6.5, 6.8, 7 7.5, and 8.05 GHz are $21.6^\circ$, $14.5^\circ$, $14.3^\circ$, $12.9^\circ$, $11.6^\circ$, $10.8^\circ$, $11.01^\circ$, $11.3^\circ$, $14.3^\circ$, $9.4^\circ$ and $8.25^\circ$, respectively. The simulated and measured normalized gain patterns agree well, except for a few discrepancies due to some faults in the measurement setup and variations in distances from the RA to the feeding waveguide, or reference antenna. The primary cause of the fluctuation in side lobes may be attributed to the phase changes of the overall electric field profile in the array, particularly in the higher frequency range. In addition, the AR keeps decreasing as the magnitude of one of the orthogonal components of the electric field becomes higher than the other, violating the condition of achieving CP. This phenomenon can be justified as antenna gain is increasing with the increasing AR value.

## Active feeding strategy with corresponding beamsteering performance

**Fig. 6:**
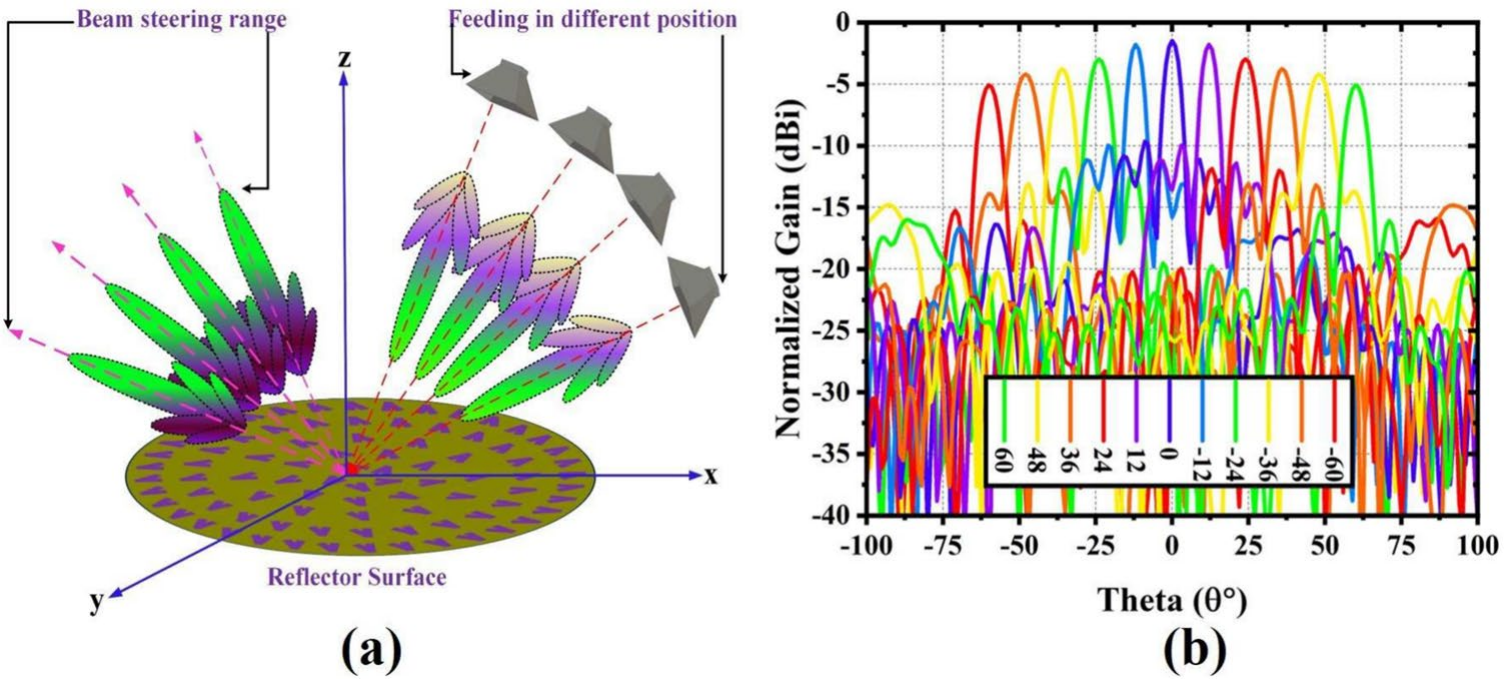
Proposed based beam-steering (**a**) strategy by changing the location of the feeding horn antenna positions and (**b**) different scan angles or positions of the active feeding or horn antenna at 7.78 GHz (Elevation plan).

The RA main beam’s versatility can be expanded to an entirely new level if it can be steered in different directions. Additionally, the coverage area can be expanded by steering the beam in various directions. Conventionally, a reflectarray is physically rotated, or the feeding position is changed to perform beamsteering, which is known as active or mechanical beam steering^19^. The beam steering of any antenna array is possible through electrical beam steering using active components such as diodes, capacitors, or other active components or structures, etc, or by mechanically moving the antenna or changing the feeding positions. However, electrical beam steering reduces weight, achieves better control,and flexibility; its high complexity and fabrication cost render this technique undesirable. In this scenario, beam steering using an active feeding technique is considered as a promising solution. Figure 6a,b show the active feeding strategy and the corresponding steering performance. Initially, the RA position with respect to the waveguide or reference antenna is considered as a $0^\circ$ or $90^\circ$ elevation ($\theta$) plane. The distance between the feed antenna and the RA is also the same, and the position of the feeding antenna is varied $\theta$ planes for different angles such as $12^\circ$, $24^\circ$, $36^\circ$, $48^\circ$ and $60^\circ$ and $-12^\circ$, $- 24^\circ$, $- 36^\circ$, $-48^\circ$ and $-60^\circ$ for $90^\circ$ so that it creates $12^\circ$, $24^\circ$, $36^\circ$, $48^\circ$ and $60^\circ$ and $-12^\circ$, $- 24^\circ$, $- 36^\circ$, $-48^\circ$ and $- 60^\circ$ elevation or $\theta$ plane. Due to the achieved CP polarization properties at 6 GHz of the RA, the mechanical beam steering is analyzed and performed for this frequency. The scanning performance shows that the achieved beam steering range is ± $60^\circ$ in $\theta$-plane. Due to the $12^\circ$ difference in feeding positions for each scanning performance, the achieved beam steering angle difference from one beam to another beam is also $12^\circ$. The beam is steered on the right side $60^\circ$ and on the left side $- 60^\circ$ with respect to the different $0^\circ$ positions of the feeding horn on the surface of the RA antenna in the elevation plane. It should be noted that the scanning loss is observed to be around 4 dB, which occurs due to the misalignment or displacement of the feeding antenna for every scanning step.

**Table 3:** Comparison with other state-of-the-art works.

Refs.	Size ($\hbox {mm}^2$)	Freq. (GHz)	BW (%)	Gain (dBi)	Layer	Effi. (%)	Scaning range	Polarization
^20^	$175\times 175$	6,12	33, 40	17.7, 24.3	1	39, 43	$0^\circ$	CP
^21^	$119\times 119$	30	17.48	29	1	5.524	± $40^\circ$	Linear
^14^	$730\times 600$	10.7–12.75	6.77	12.5	2	5	± $15^\circ$	Linear
^22^	*D* = 415	8.23	*N*/*A*	25.75	1	63	$0^\circ$ to $50^\circ$	Linear
^23^	$250\times 250$	9.5–10.5	8	25	4	75.7	*N*/*A*	Linear
^24^	$200\times 200$	12	8–10	25.5	1	43.1	± $21^\circ$	CP
^25^	$700\times 300$	14	*N*/*A*	25.1	1	70	± $45^\circ$	*N*/*A*
^26^	$215\times 215$	15–24	24.5	25.1	1	49.93	± $35^\circ$	CP
^27^	$215\times 215$	8–12	40	30.6	3	40	± $45^\circ$	Linear
^28^	$832\times 806$	12.8–14.2	10	22	3	40	± $27.5^\circ$	Linear
Proposed	$D=259.65$	5–10	63.11	21.26	2	54.75	± $60^\circ$	CP/Linear

$RA.^*$ reflectarray, $CP.^*$ circular polarization, $Effi.^*$. efficiency $D.^*$ diameter.

The performance of the proposed reflectarray is compared with the other state-of-the-art reflectarrays in Table 3. Most of the prior works have used reflectarrays with a higher footprint (i.e., about 3*X*) dimensions compared to the proposed design. In^28–31,35,36^, the authors have presented reflectarrays with linear polarization. On the contrary, the proposed antenna has achieved circular polarization. Though in^27,28,30−35^, the reflectarrays show a bit higher gain, the proposed reflectarray shows higher fractional bandwidth with a moderate gain of 22 dBi. The proposed ultrawideband *Y*-shaped element-based reflectarray can achieve not only the better BW and efficiency of $63\%$ (fractional BW) and $54.75\%$ but also the highest scanning range of ± $60^\circ$ compared to all the reported works. From Table 3, it is also apparent that our proposed reflectarray shows a good trade-off between the fractional bandwidth, gain, efficiency, scanning range, and polarization property.

**Fig. 7:**
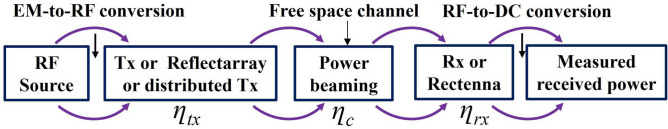
The three components of efficient wireless power transfer over a distance, each playing a critical role in the overall functionality of the WPT system.

## The demonstration of the wireless power transfer (WPT) system for different case scenarios

The primary distinction between wireless power transfer (WPT) systems and communication systems lies in the efficiency of power transfer. Typically, communication systems capture signals from all directions as transmitters disperse them widely, while WPT systems concentrate on a single receiver or specific receivers to achieve efficient power transfer. As a result, although communication systems receive sufficient signal power for communication purposes, their efficiency is much lower compared to WPT systems. Therefore, efficiency is a crucial factor in the design of WPT systems. The efficiency of the first block (as shown in Fig. 7), which is the electromagnetic (EM) wave to RF conversion efficiency is equal to the array antenna efficiency ($\eta _{tx}$). The efficiency of the transmitting antenna indicates its capability to radiate the RF power supplied by the RF generator into the free space. The second component is the free space channel, where the RF input power radiated from the array antenna is transferred through a focused beam across free space to reach a receiver. To achieve maximum beam collection efficiency, it is essential to choose an optimal power density distribution across the transmitting antenna aperture. A non-uniformly illuminated aperture enhances beam collection efficiency, and research indicates that the ideal taper shape is Gaussian^29^ Goubau’s relation describes a design parameter as being directly related to the beam collection efficiency^30^.

$$\begin{aligned} \tau =\frac{\sqrt{(A_{rx}A_{tx})}}{\lambda _0 D} \end{aligned}$$

where $A_{rx}$ and $A_{tx}$ are the aperture areas of the receiver and transmitter antennas, and the distance between them is *D*. Here $\tau$ is the reflection coefficient of the antenna. As evident from this equation, Goubau’s relation is applicable for calculating the dimensions of the apertures involved. The beam collection efficiency ($\eta _c$) is calculated using the following equation^30^:

$$\begin{aligned} \eta _c= \left( 1-e^{-\tau ^2} \right) \times 100\% \end{aligned}$$

This equation scales up with the power density and the increased aperture area of the antenna. For instance, as the area $A_{tx}$ increases, the incident power density also increases, resulting in an improved beam collection efficiency, demonstrated. This reflects a trade-off between the efficiency and aperture size of the TX and RX antennas. The total efficiency ($\eta _{toll}$) of a WPT system is defined as the ratio of the DC output power at the receiver to the DC input power at the transmitter, expressed as^31^

$$\begin{aligned} \eta _{toll}=\eta _{tx}\eta _c\eta _{rx} \end{aligned}$$

This implies that the end-to-end efficiency encompasses all the component efficiencies from the DC output power supply feeding the RF source in the transmitter to the DC power interface at the receiver output.

**Fig. 8:**
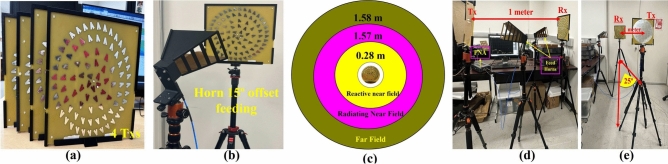
(**a**) Four RA (Tx) enclosed with plastic frames, (**b**) alignment and position of the TX antenna using a tripod stand (elevation plan), (**c**) different field region of the TX based on the 3.5 GHz frequency band, (**d**) Side view of the WPT system demonstration (**a**) distance between one Tx and one Rx is 1 m enclosed with plastic frames, and (**e**) straight view for similar setting to realize the alignment between the Tx and Rx antenna.

Figure 8a–c depict the reflectarray system utilizing a $15^\circ$ offset feeding technique and different field regions at a frequency of 3.5 GHz. Here are three regions to consider: the reactive near field (up to 0.28 m), the radiating near field (from 0.28 to 1.57 m), and the far field (1.58 m and beyond). These regions account for the aperture area of the Tx or Rx and the selected frequency band. The 3.5 GHz frequency band is chosen due to the better transmission coefficient, minimizing far-field region and path loss. In order to achieve maximum power transfer efficiency, the WPT system is demonstrated within the near-field region or the radiating near-field region. The distances between Tx and Rx antenna such as 1, 1.7, and 4.06 m represent the radiating near-field distances for one, two, and three Tx systems, respectively. Based on the aforementioned beam collection and overall efficiency of the WPT system, the proposed WPT system is evaluated for the following case scenarios

- One Tx and one Rx with 1 and 1.7 m distance between them.
- Two Txs and one Rx with a 1.7 m distance between them.
- Three Txs and one Rx with 4.064 m distance between them.

**Table 4:** Theoretical and measured transmission coefficients for frequency bands and power transfer efficiency (PTE), when one TX and Rx distance is 1 m.

Frequency (GHz)	Simulated $S_{21}$ $(dB)$	Measured $S_{21}$ $(dB)$	PTE ($\%$) Sim./Mea.	Frequency (GHz)	Simulated $S_{21}$ $(dB)$	Measured $S_{21}$ $(dB)$	PTE ($\%$) Sim./Mea.
2.89	12.21	12.59	5.5/6.0	3.66	10.81	12.34	8.29/5.83
3.31	12.11	12.60	6.14/5.49	5.41	18.20	14.41	1.50/3.62

### One Tx and one Rx WPT system

**Fig. 9:**
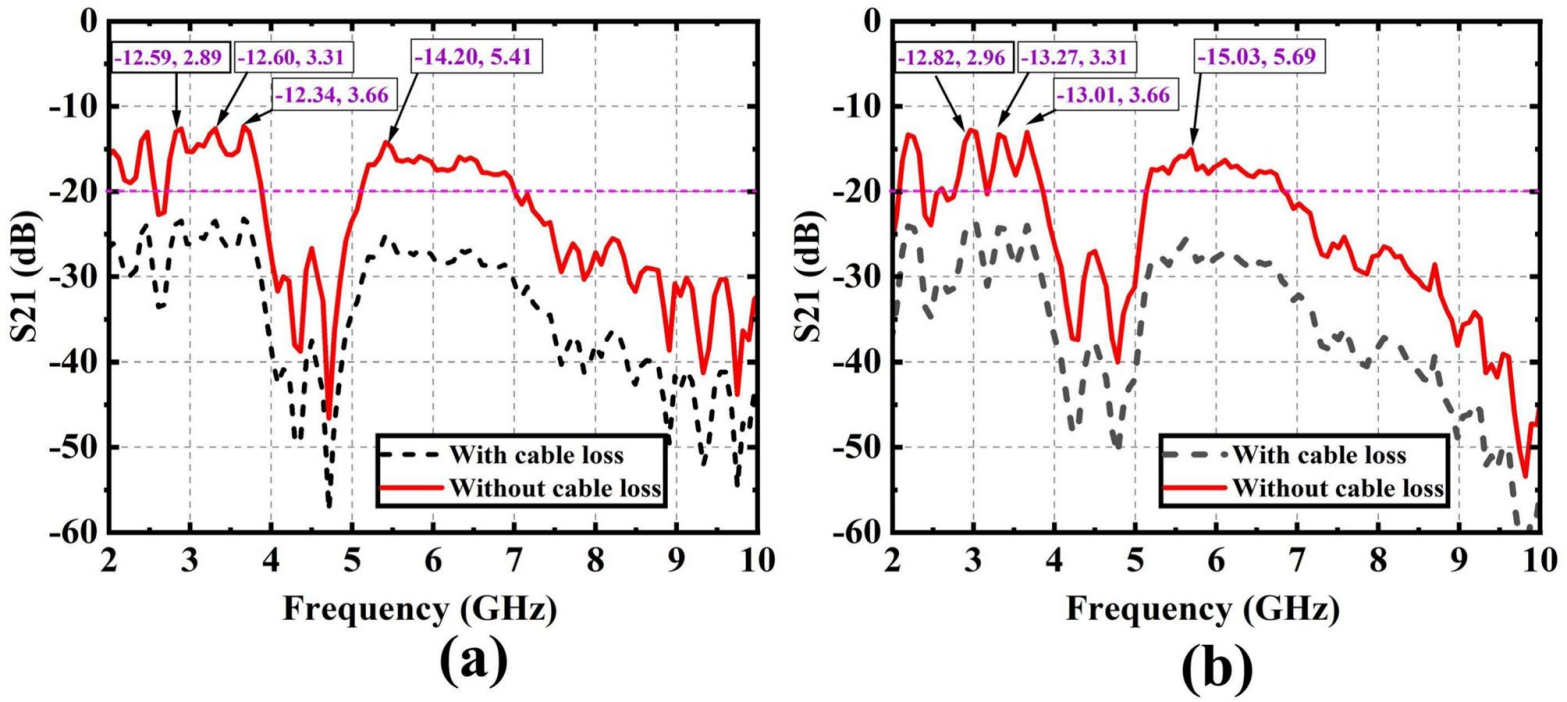
Transmission coefficient $S_{21}$ (**a**) between one Tx and one Rx is apart from 1 m distance, and (**b**) between one Tx and one Rx is apart from 1.7 m distance.

First, two scenarios are analyzed based on the transmission coefficient for the same reflectarray serving as both Tx and Rx. Figure 8d,e depict the setup of a single Tx and single Rx WPT system in a laboratory environment, including a side view. The distance between Tx and Rx is 1 m, which is within the region of radiating near the field region. Figure 9a,b show the transmission coefficient ($S_{21}$) between one Tx and one Rx is apart from 1 and 1.7 m distance, respectively. The measured transmission coefficient in both cases includes cable loss. The total cable loss for the first case (i.e., 1 m distance with one tx and one Rx system) in this demonstration is 9.7 dB. Table 4 presents the theoretical and measured values of the transmission coefficient with 1 distance for various frequency bands, along with their corresponding power transfer efficiency (PTE). The measured transmission coefficients without cable loss in this demonstration are 12.59, 12.60, 12.34, and 14.20 dB for the frequency bands of 2.89, 3.31, 3.66, and 5.41 GHz respectively. Meanwhile, the theoretical transmission coefficients are 12.21, 12.11, 10.81, and 18.20 dB for the same frequency bands, respectively. The power transfer efficiency (PTE) is calculated by the following equation from the simulated and measured transmission coefficients^32^.

$$\begin{aligned} PTE(\%) =10^\frac{S_{21}}{10}\times 100 \end{aligned}$$

The measured PTE at different frequency bands (i.e., 2.89, 3.31, 3.66, and 5.41 GHz) for $6\%$, $5.49\%$, $5.83\%$, and $3.62\%$, while the simulated PTE are $5.5\%$, $6.14\%$, $8.29\%$, and $1.50\%$, respectively. The same arrangement is realized using a Keysight MXG analog signal generator (*N*5183*B*) with a frequency range of 9 kHz to 40 GHz for RF input. The frequency band and the corresponding output power levels are analyzed by a Keysight MXG signal analyzer (N9020B). For a 24 dBm RF input, the received power with cable loss (11.05 dB) is 22.82, 21.85, 23.60, and 29.60 dBm at the 3.38, 3.31, 3.66, and 5.41 GHz frequency bands, respectively. The achieved PTE is $5.22\%$, $5.49\%$, $5.55\%$ and $3.50\%$ at the 3.38, 3.31, 3.66, and 5.41 GHz frequency bands, respectively. The achieved PTE for several cases, such as the simulated and measured transmission coefficient and the measurements from the signal analyzer are in good agreement, except for minor discrepancies. The discrepancy between theoretical or simulated and measured PTE can arise due to differences in how these powers are modeled in the simulation versus how they are actually measured in the experiment. In any real system, there are losses that are often difficult to fully account for in a simulation. These include (1) conductor Losses: The simulation might use idealized components (i.e., no resistance), which would predict higher efficiency. In reality, the connected wires in the system have resistance and power is dissipated as heat due to Joule heating. Quantify this difference by introducing a resistance term in the simulation

$$\begin{aligned} PTE_{simulated}=\frac{P_{out}}{P_{in}+P{loss}} \end{aligned}$$

where $P_{loss}$ = $I^2R$, and the resistance might be underestimated in the simulation. Similarly, in simulation it is assumed in perfect Magnetic Field Coupling (e.g., alignment fields), which leads to higher predicted efficiency, but in practice, misalignment or imperfect coupling causes a loss in power transfer. Quantify misalignment by introducing a coupling coefficient k in the simulation. The power transfer efficiency is related to this coefficient:

$$\begin{aligned} PTE \sim {k^2} \end{aligned}$$

If the coupling coefficient is less than ideal in the experiment, it should be accounted for and compared with the simulation value. (2) Path Losses (Coupling and Radiation Losses): The simulation might not fully account for energy losses due to radiation or imperfect near-field coupling, particularly in systems like wireless power transfer (WPT). In measurement, the path losses can be significant in a real-world setup, especially over larger distances or with non-ideal materials. The power transfer efficiency can be impacted by the distance between transmitter and receiver, geometry, and frequency. A typical model for path loss (in free space for example) is:

$$\begin{aligned} PTE \sim \frac{1}{d^2} \end{aligned}$$

where *d* is the distance between the antennas ($T_x$ or $R_x$). In the simulation, this might be idealized (e.g., near-field losses ignored), leading to an overestimation of PTE compared to the real-world setup. (3) Power Conversion Efficiency: The simulation might assume perfect power conversion at the transmitter and receiver (e.g., no losses in amplifiers, rectifiers, etc.), where in measurement power conversion efficiency is not perfect. Quantify this difference by adjusting the conversion efficiency in the simulation model:

$$\begin{aligned} PTE_{simulated}=\frac{\eta _{conversion}.P_{out}}{P_{in}} \end{aligned}$$

where $\eta _{conversion}$ is the efficiency of power conversion in the transmitter or receiver. If this is lower in the experiment than in the simulation, it would explain a lower measured PTE. (4) Measurement Errors: The simulation assumed ideal conditions for power input and output measurements with no error or noise, where in measurement errors such as sensor calibration issues, signal interference, or noise can lead to discrepancies in recorded power levels. Quantifying the sources of discrepancy is another reason for data mismatch. There are also several factors such as Imperfect alignment and power conversion efficiency that led to discrepancy between theoretical or measured data.

Table 5 displays the theoretical and measured transmission coefficient values across various frequency bands, along with their corresponding PTE. The measured transmission coefficients without cable loss in this demonstration are $- 12.82$, $- 13.72$, $- 13.01$, and $- 15.03$ dB for the frequency bands of 2.96, 3.31, 3.66, and 5.69 GHz, respectively. Meanwhile, the theoretical (i.e., simulated values) transmission coefficients are $- 16.86$, $- 16.72$, $- 15.42$, and $- 22.81$ dB for the same frequency bands. The achieved measured PTE values are $5.22\%$, $4.70\%$, $5.00\%$, and $3.14\%$, while the simulated PTE values at the 2.96, 3.31, 3.66, and 5.69 GHz frequency bands are $2.05\%$, $2.12\%$, $2.86\%$, and $0.52\%$, respectively.

**Table 5:** Theoretical and measured transmission coefficients for frequency bands and power transfer efficiency (PTE), when one TX and Rx distance is 1.7 m.

Frequency (GHz)	Simulated $S_{21}$ $(dB)$	Measured $S_{21}$ $(dB)$	PTE ($\%$) Sim./Mea.	Frequency (GHz)	Simulated $S_{21}$ $(dB)$	Measured $S_{21}$ $(dB)$	PTE ($\%$) Sim./Mea.
2.96	16.86	12.82	2.05/5.22	3.66	15.42	13.01	2.86/5.00
3.31	16.72	13.72	2.12/4.70	5.69	22.81	15.03	0.52/3.14

**Fig. 10:**
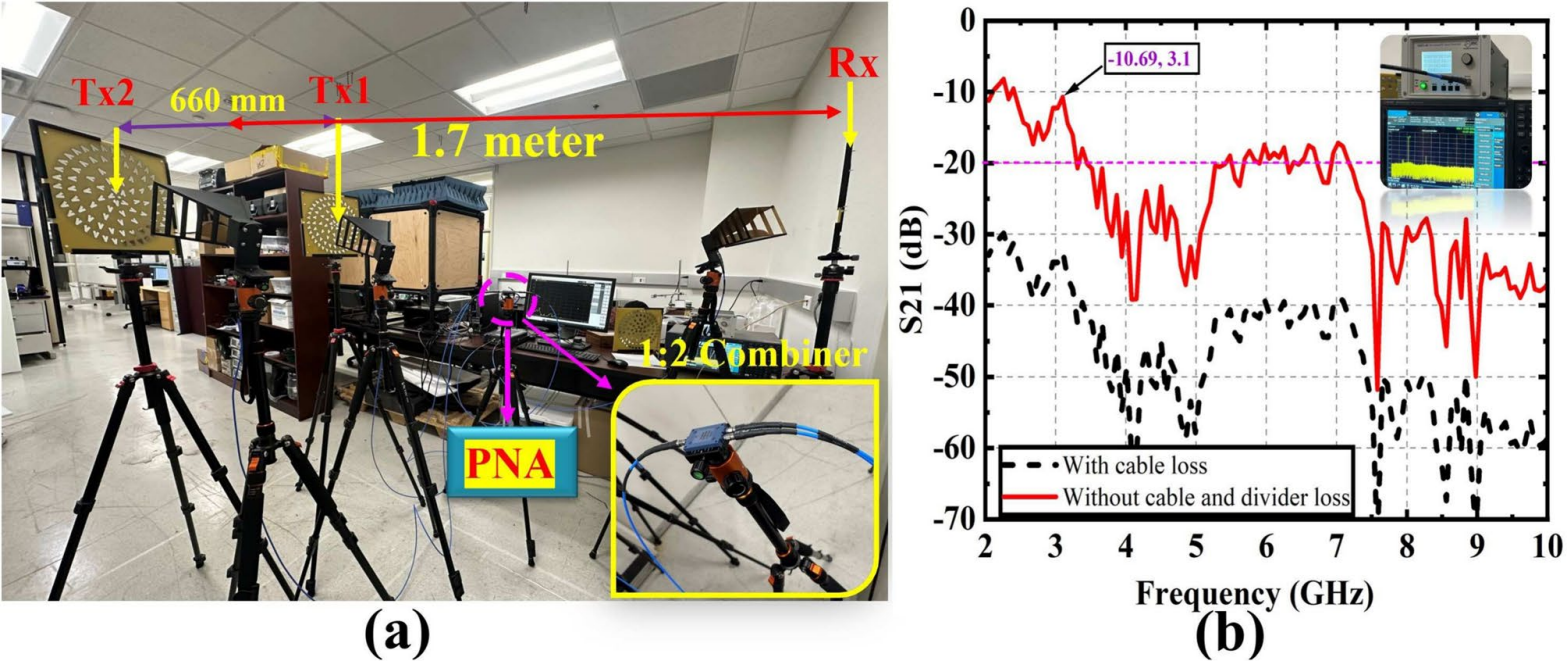
(**a**) Setup in one Tx and one Rx between 1.7 m distance, and corresponding (**b**) transmission coefficient $S_{21}$.

### Two Tx and one Rx WPT system

Figure 10a,b show the WPT demonstration where the distance between two Tx and one Rx is 1.7 meter and the corresponding transmission coefficient. The distance between Tx1 and Tx2 antennas is 660 mm. The distance is determined based on the optimal transmitted power from the Tx end. This separation is verified by measuring $S_{21}$. Various separation distances were observed to find the optimal values of $S_{21}$. The PE2092 2 way type N power divider is used to connect Tx1 and Tx2 antennas with insertion loss of 9.35 dB. The transmission coefficients (without loss) in this arrangement are 9.21 and 10.69 dB at 2.25 and 3.10 GHz, respectively. The total loss (including the power divider and cables losses) in this demonstration is 21.75 dB. The achieved PTE for two transmitters and one receiver system is $11.90\%$ and $8.53\%$ at 2.25 GHz and 3.10 GHz, respectively. The same arrangement is realized using a signal generator for the RF input and a signal analyzer for analyzing the output. The received power (with cable losses) are − 24.03, 24.87, 24.55 and 26.29 dB at 2.96, 3.31, 3.66, and 5.69 GHz frequency bands, respectively. The cable loss in this scenario is 11.05 dB. The achieved PTE at similar frequency bands are $5.03\%$, $4.14\%$, $4.46\%$, and $2.99\%$, respectively. Similarly, the achieved PTE for 20 dBm RF input power in a system with two TXs and one Rx is $11.20\%$ and $8.33\%$ at the same frequency bands of operation. It is noticeable that at the same distance (1.7 m), the achieved PTE between one Tx and two Tx WPT systems are $4.70\%$ and $8.53\%$ at the 3.31 GHz frequency band. This happened due to the higher transmitted power in the two Tx systems compared to the one Tx system. It is that the combined gain of the two Txs is much higher than the single TX which is why the PTE is higher. Therefore, the distributed transmitter (Tx) system is a promising candidate for a long-distance wireless power transfer (WPT) system. The theoretical limit of Power Transfer Efficiency (PTE) in a two-transmitter Wireless Power Transfer (WPT) system at a distance of 1.7 m can be estimated using the Friis transmission equation or the near-field and far-field coupling models in WPT systems. More specifically details about the system, such as the frequency of operation, the alignment of transmitters and receivers, and the specific characteristics of the WPT technology in use (e.g., resonant inductive coupling, capacitive coupling, or other forms of wireless power transfer). In a two-transmitter WPT system, the power transfer efficiency is affected by factors such as distance, where the power is decreased with the square of the distance (in free space). Secondly, the wavelength of the electromagnetic waves, which depends on the operating frequency, influences the efficiency. Thirdly, the alignment, shape, and tuning of the antennas can drastically influence efficiency and finally, environmental factors like interference, obstacles, and material properties of the medium also impact efficiency.

**Table 6:** Comparison between the proposed rectifier with other state-of-the-art works.

Refs.	Frequency (GHz)	Input power (dBm)	Maximum PCE (%)
^33^	2.5	5	70
^34^	3.4	10	54.4
^35^	2.5	14	75.8
^36^	5.4	13	75.6
^37^	2.1	14	76
^38^	2.35	13	80.4
This work	2.55, 3	11	82, 71

**Fig. 11:**
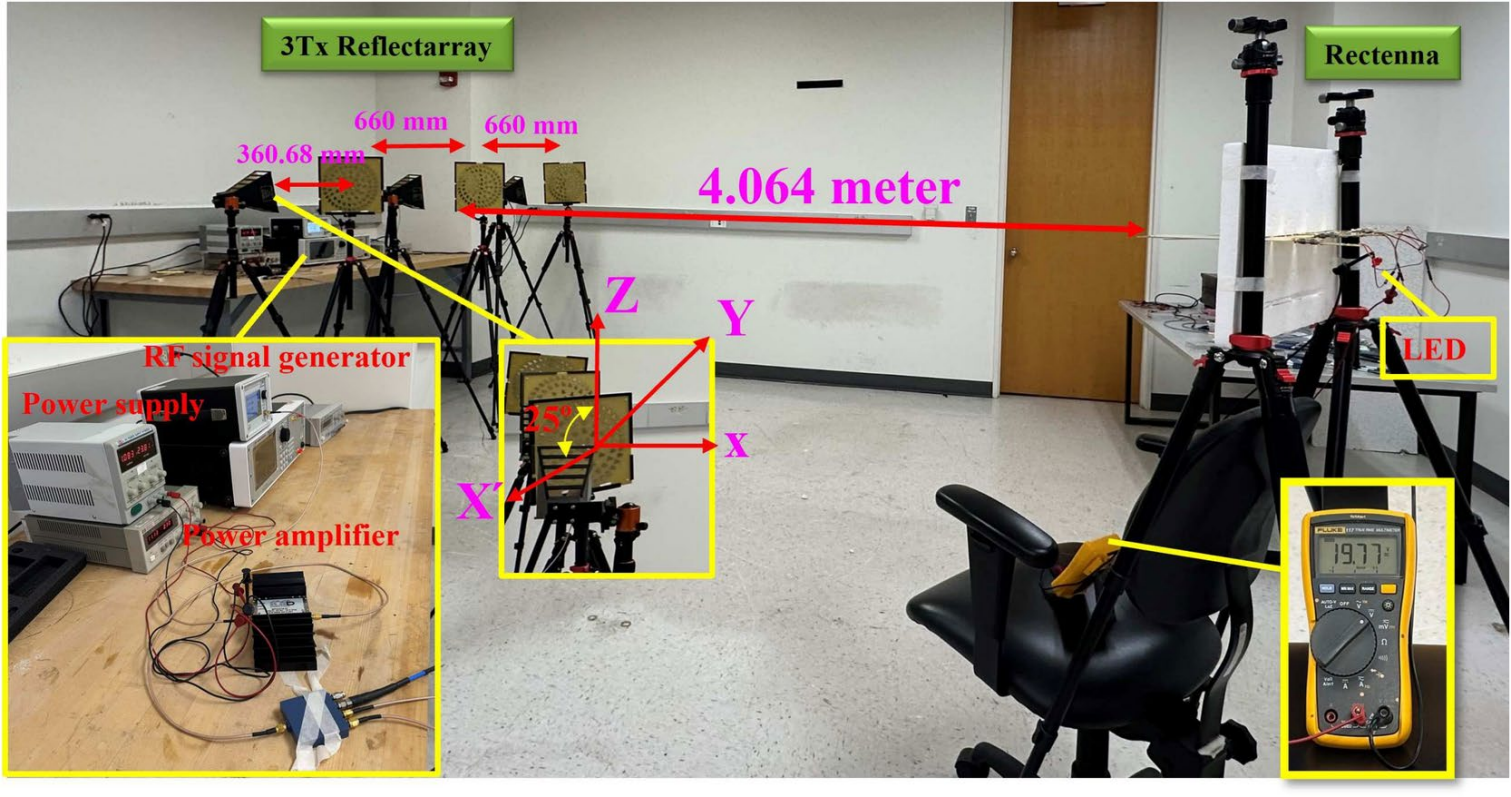
WPT system demonstration using 3Tx and one Rx system with a distance of $4 \,\hbox {mm}$.

**Table 7:** Comparison between achieved wpt system performance with other state-of-the-art works.

Ref.	Size ($\hbox {mm}^2$)	Freq. (GHz)	Dis. (m)	Effi. (%)	Justification
^39^	$210\times 210\times 40$	5.8	10–15	0.0034, 0.0012	Measured
^40^	$272\times 272\times 280$	5.2	1–4	0.156, 1.79	Measured
^41^	$272\times 272\times 280$	5.2	1–3	0.058, 1.19	Measured
^42^	$260\times 380\times 88$	5.75	4.2	0.2	Measured
^43^	$63\times 63$	1.5	0.055	0.473	Measured
^44^	$63\times 63$	1.5	0.04	0.1	Measured
^45^	$8\times 4\times 1.6$	2.4	0.3	0.001	Measured
^46^	$340\times 330$	2.4	0.42	2.7	Measured
^47^	$60\times 60$	1.47	0.05	0.67	Measured
Proposed	$D=259.65$	3, 3.5	4.064	8.11	Measured

### Three Txs and one Rx with a distance of 4 m

Figure 11 shows a demonstration of the WPT system where 3 Txs are aligned in such a way that they collectively maximize the transmitted power to the Rx end. The selected distance is chosen based on the maximum beam collection efficiency ( as discussed in section V), taking into account both the Tx and Rx aperture dimensions. Table 6 presents a performance comparison of current state-of-the-art works. The proposed rectifier outperforms these recent advancements. It achieves higher a power conversion efficiency (82% and 71%) at low RF input power and operates in dual bands (2.55 GHz and 3 GHz).

Each Tx is fed through a horn antenna placed 360.68 mm from the reflecting surface, with a $25^\circ$ offset position. The separation between each Tx is 660 mm. This optimized separation between each Tx ensures the optimal transmitted power to the rectenna end. The distance between three Txs and Rx is 4 m. The distance between the Tx and Rx is optimized to ensure that the Rx receives the maximum power and get the $100\%$ beam collection efficiency. The RF signal generator is used to provide the minimum level of RF input power and frequency band for boosting the RF level, which serves as the input power for each Tx system. The RF input power and frequency band provided by the signal generator are 4 dBm ($p_1$ dB point) and 3 GHz, respectively. The Microwave Dynamics amplifier (model no. MPA0218-37), with a frequency range of 2 to 18 GHz, is used for the WPT system. Based on the RF input power, the boosted amplified RF level is 34.34 dB with $52\%$ power added efficiency, which is the RF input for each Tx system. The cable and power divider loss in this arrangement is 4.75 dB. The radiation efficiency of each Tx system is $54.5\%$. Therefore, the exact resultant Tx output is 5.37 dBm or 3.44 mW. The resultant generated voltage at the Rx end is 19.77 volts or 27.91 mW, where the total load at the RX end is $1.4\,\hbox {k}\Omega$. The achieved PTE for this demonstration is $8.11\%$. The main limitation in this demonstration is that a single power amplifier is used to power the three Txs systems through a power divider. Due to this limitation, the resultant Tx power is not sufficient to transmit a high amount of EM signal over long distances. For this demonstration, it is noticeable that the achieved PTE of the three Txs system is better than that of a single Tx system.

**Fig. 12:**
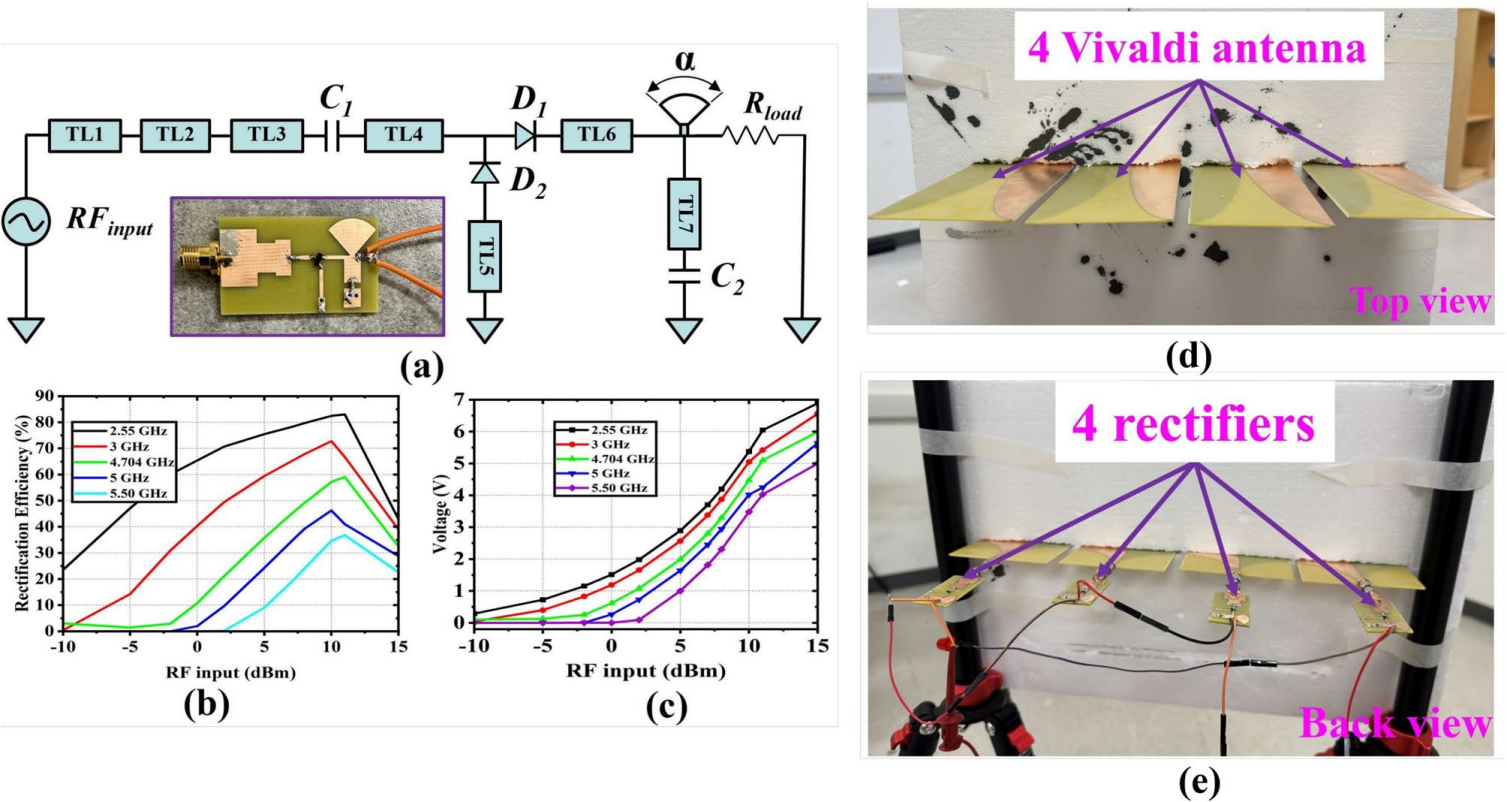
(**a**) Schematic of the proposed rectifier and its prototype, (**b**) rectification efficiency for different frequency bands with various RF input power levels, and (**c**) generated DC output voltage for different frequency bands with various RF input power levels. Proposed $4 \times 1$ rectenna array: (**d**) top or front view of the array, and (**e**) back view of the array showing the DC line connected by wires.

Figure 12a shows the proposed rectifier and corresponding prototype fabrication. The circuit is modeled and evaluated using Keysight advanced design system (ADS) software. It includes a matching network, a DC block capacitor, a Schottky diode in a voltage doubler configuration, a DC pass filter capacitor, and a load. The voltage doubler setup uses the *HSMS*-2860 Schottky diode, package in the *SOT*-23 package. The design utilizes an FR4 substrate with a thickness of 1.524 mm. The broadband matching network comprises a short circuit stub in series with a shunt diode in the voltage doubler (VD) configuration, along with three series transmission line (TL) segments. The input impedance ($Z_b$) of the shunt stub in series with the diode ($D_2$) is calculated as^48^:

$$\begin{aligned} Z_b =\frac{(Z_a+Z_D).(Z_{dcfilter}+Z_D)}{Z_a+2Z_D+Z_{dcfilter}} \end{aligned}$$

here $Z_{dcfilter}$ is the input impedance seen across the DC pass filter, while $Z_D$ represents the impedance of each diode. According to the characteristics of the DC pass filter, $Z_{dcfilter}$ is infinite at all frequencies. The impedance $Z_a$, given by $jZ_{oTL_1}$ $tan(\beta LTL_1)$, of the short circuit stub is used to compensate for the imaginary part of $Z_D$ from the lower ($f_{Lo}$) and upper ($f_{up}$) cutoff frequency bands, respectively. Therefore, $Z_b$ is dependent on both frequency and input power ($P_in$) due to the nonlinear behavior of the rectifying diode and can be re-expressed as shown in the following^49^:

$$\begin{aligned} Z_b = Z_D + jZ_{oTL_1}tan(\beta LTL_1) \end{aligned}$$

The value of TL has been tuned to achieve Re[$Z_{in}$($f_{center}$)] $\approx$ 50 $\Omega$ and Im[$Z_{in}$($f_{center}$)] $\approx$ 0 $\Omega$. The rectifier’s input impedance is chosen to be nearly equal to one on the Smith chart. When the parameters are optimized, the rectifier exhibits multiple frequency bands where the magnitude of $S_{11}$ is less than $- 10$ dB, with the input impedance for each band being approximately 50 $\Omega$. The optimized dimensions of the different transmission lines and radial stubs are in mm as shown in Fig. 12a: $L_{TL1}$ = 9.926, $W_{TL1}$ = 13.63, $L_{TL2}$ = 5, $W_{TL2}$ = 7, $L_{TL3}=4.2,$ $W_{TL3}$ = 8.6, $L_{TL4}$ = 4.8, $W_{TL4}$ = 1, $L_{TL5}$ = 7.88, $W_{TL5}$ = 1.04, $L_{TL6}$ = 6.2, $W_{TL6}$ = 0.9, $L_{TL7}$ = 5.797, $W_{TL7}$ = 4.69, and $L_{rad}$ = 8.69, $W_{rad}$ = 1, $\alpha _{rad}$ = $90^\circ$, $C_1 = C_2$ = 27*pF*. The rectifier circuit is initially simulated using harmonic balance and large-signal scattering parameters analysis in Keysight ADS. Due to its superior efficiency at the input power level of 0–10 dBm, a 3.5k $\Omega$ resistor is chosen as the load. Figure 12b illustrates the relationship between frequency and power conversion efficiency (PCE) at RF input levels of $- 10$ to 15 dBm. The measured PCE exceeds $50\%$ at $- 2$ dBm for 2.55 GHz. For 0 dBm RF input power, the achieved PCEs are $65\%$ and $40\%$ at 2.55 GHz and 3 GHz, respectively. For 11 dBm RF input power levels, most of the frequencies showed their maximum PCE. From the 2.55 to 4.704 GHz band, they achieved more than $55\%$ PCE, while the 5 to 5.5 GHz band achieved more than $33\%$ PCE. It is notable that for 11 dBm RF input power, the maximum PCE is achieved at 2.55 GHz and 3 GHz, with values of $82\%$ and $71\%$, respectively. Figure 12c illustrates the relationship between Vout and RF input power levels for different frequency bands. It shows that for most frequency bands, more than 4 volts is achieved at 11 dBm RF input power. For 0 dBm RF input power, the generated DC voltages at 2.55 GHz and 3 GHz is more than 1 *V*. It can be concluded that based on the achieved PCE and generated voltage at different RF input power levels across are several frequency bands, the proposed rectifier is a good candidate for the WPT system. Figure 12d,e show the linear rectenna array top and back view, respectively. The typical Vivaldi antenna is used as the receiving antenna, operating from 2 to 12 GHz with a gain of 7 dBi for a single antenna element. Four antenna elements are directly connected to four rectifiers with a spacing of $\Lambda /2$ for 4 GHz frequency band, with the DC lines of each rectifier connected in series to realize the resultant generated voltage. Table 7 shows the comparison between the achieved PTE of the proposed WPT system and some existing WPT systems. For reference^35–37^, the achieved PTE and distance between Tx and RX is better than the proposed system, but their aperture area is larger than the proposed Tx system. For reference [38], the achieved PTE is $45\%$ at 10 m distance, while the proposed WPT system achieved PTE is $8.11\%$ at 4.064 m distance. So, it can be concluded that based on the aperture area and PTE, the proposed distributed WPT systems is a promising candidate for long-distance power beaming.

## Conclusion

In summary, this paper presents a multilayer RA antenna that can solve the phase range or spatial delay problem of single-layer RA. The feeding technique is also improved by giving general specifications for RA for offset-feeding scenarios. Using the new RA antenna for the WPT system in conjunction with a distributed TX system is a promising approach for long-distance power beaming. The Power Transfer Efficiency (PTE) achieved for various distributed Wireless Power Transfer (WPT) configurations-comprising one, two, and three transmitters paired with a single receiver-was $5.03\%$, $8.33\%$, and $8.11\%$, respectively, at a distance of 1.7 m, with an antenna spacing of 774.7 mm. Several experimental scenarios demonstrate that the distributed TX system can transmit more power to the RX end and more efficiently than a single TX-RX WPT system. This research addresses the critical need for efficient, long-distance WPT systems by introducing significant advancements in wireless energy applications. The innovative system design, featuring optimized transmitter configurations and enhanced antenna structures, improves power transfer efficiency at extended distances. These advancements highlight the potential for more effective wireless power solutions. Overall, the design and performance position this system as a substantial contribution to the field of wireless power technology.

## Data Availability

The datasets used and/or analyzed during the current research are available from the corresponding author upon reasonable request. The data has been provided as supplementary material.
